# *Candidatus* Liberibacter asiaticus: An important factor affecting bacterial community composition and *Wolbachia* titers in Asian citrus psyllid

**DOI:** 10.3389/fmicb.2023.1109803

**Published:** 2023-02-07

**Authors:** Rui-Xu Jiang, Feng Shang, Hong-Bo Jiang, Wei Dou, Tomislav Cernava, Jin-Jun Wang

**Affiliations:** ^1^Key Laboratory of Entomology and Pest Control Engineering, College of Plant Protection, Southwest University, Chongqing, China; ^2^International Joint Laboratory of China-Belgium on Sustainable Crop Pest Control, Academy of Agricultural Sciences, Southwest University, Chongqing, China; ^3^Institute of Environmental Biotechnology, Graz University of Technology, Graz, Austria

**Keywords:** *Diaphorina citri*, endosymbionts, *Wolbachia*, 16S rRNA gene sequencing, citrus HLB

## Abstract

Endosymbionts play crucial roles in various physiological activities within insect hosts. The Asian citrus psyllid (ACP), *Diaphorina citri* Kuwayama, is an important vector for *Candidatus* Liberibacter asiaticus (*C*Las), a fatal pathogenic bacterial agent causing the disease Huanglongbing in the citrus industry. This study combines high-throughput sequencing of 16S ribosomal RNA amplicons to explore how *C*Las affects the bacterial community in different color morphs (blue, gray), genders, and tissues (cuticle, gut, mycetome, Malpighian tubule, ovary, and testis) of ACP. We found that there was no significant differences in the bacterial community diversity and *C*Las acquired ratio between the different color morphs and genders of ACP adults. However, acquiring *C*Las could promote the adult bacterial community’s diversity and richness more than in the uninfected condition. The presence of *C*Las could increase the *Wolbachia* and unclassified_*Enterobacteriaceae* proportions more than in the uninfected condition. The bacterial community diversity in the *C*Las infected tissues of ovary and cuticle, was lower than the uninfected condition, but the richness of all tissues was not different between the infected and uninfected conditions. *C*Las could also change the bacterial structure in different tissues and make the bacterial relationship network simpler than it is in an uninfected condition. Furthermore, we used quantitative real-time PCR to assess the dynamic changes of *Wolbachia* in *C*Las uninfected and infected color morphs and tissues of ACP. The results showed that *Wolbachia* titers were significantly higher in *C*Las infected adults than in uninfected adults. In different tissues, the *Wolbachia* titers in the testis, ovary, and Malpighian tubule were higher than their uninfected counterparts. Our results provide essential knowledge for understanding the symbionts of the ACP and how *C*Las affects the bacterial community of the ACP.

## 1. Introduction

The Asian citrus psyllid (ACP), *Diaphorina citri* Kuwayama (Hemiptera: Psyllidae), is one of the severe pests damaging citrus orchards worldwide. It plays an important role as a natural vector of “*Candidatus* Liberibacter asiaticus” (*C*Las). This particular bacterium causes the destructive disease Huanglongbing (HLB), more commonly known as citrus greening (Bové and Barros, 2006; Pelz-Stelinski et al., 2010; Grafton-Cardwell et al., 2013). HLB-infected plants develop symptoms that include stunted growth, off-season blooming, premature fruit drop, and small, misshapen, and bitter fruit (Teixeira et al., 2005; Gottwald, 2010). HLB has become one of the most economically devastating diseases affecting citrus production, with its presence being detected in most of the citrus-growing colonies worldwide.

Insects harbor diverse bacterial communities, which have profound effects on the host. These symbionts can range from disease-causing to commensal and mutually beneficial microbes. Because of their associations with symbionts, insects, in particular, are accelerating their evolutionary and ecological diversification. Microbial symbionts are also involved in the biology of insect hosts, such as the detoxification of compounds (Kikuchi et al., 2012; Ceja-Navarro et al., 2015), defense against natural enemies (Fieck et al., 2010), promotion of heat tolerance (Engel and Moran, 2013), mediating intra- and interspecific communication (Wada-Katsumata et al., 2015), and providing nutrition to the host insect. For example, aphids feed on plant sap and rely on an endosymbiotic bacterium, *Buchnera aphidicola*, to produce essential amino acids that are limited in plant phloem (Douglas, 1998). *Rhodococcus* spp. in triatomine bugs contributes to the insect’s metabolism by synthesizing B-complex vitamins that are deficient in a blood diet (Beard et al., 2002; Moran et al., 2003). Alternatively, microbial symbionts could involve the host in transmitting pathogens (Kambris et al., 2009; Hancock et al., 2011).

The ACP has two dominant distinct abdominal color morphs: blue/green and gray/brown (Wenninger et al., 2009; Xavier et al., 2014). The blue/green morphs have shown better long-duration flight capability, higher detoxification activity, and greater reproduction abilities than the gray/brown morphs (Wenninger et al., 2009; Tiwari et al., 2013; Xavier et al., 2014; Chen et al., 2019). The gray/brown morphs had smaller pronotums, shorter wings, and lower body masses than their blue/green counterparts (Wenninger et al., 2008), while, the gray/brown morphs could have higher transmission efficiency of HLB pathogen than blue/green morphs (Hosseinzadeh et al., 2021). Previous studies demonstrated that ACP harbors three domain symbionts: *Candidatus* Carsonella ruddii, *Candidatus* Profftella, and *Wolbachia* (Nakabachi et al., 2006; Tamames et al., 2007). Carsonella has been predicted to be a nutritional symbiont, providing specific nutrition for the normal activities of ACP (Nakabachi et al., 2006; Tamames et al., 2007), *Candidatus* Profftella has been known to produce diaphorin to confer a defensive advantage (Ramsey et al., 2017; Nakabachi and Fujikami, 2019), but *Wolbachia*’s function remains unclear in ACP (Ren et al., 2016). Some studies, showed the *C*Las titer had a positive relationship with *Wolbachia* (Fagen et al., 2012), so it was an important candidate that could interact with *C*Las (Kolora et al., 2015). Furthermore, *C*Las may influence ACP development, fecundity, longevity, physiological metabolism, and immune response to pathogens (Ammar et al., 2011). Consequently, it has been unclear whether different color morphs of ACP show different bacterial community characteristics and how the *C*Las affects the bacterial community in different color morphs and tissues of ACP. Therefore, our study objective was to perform 16S ribosomal RNA sequencing and downstream analysis to examine *C*Las uninfected and infected color morphs and tissues of ACP’s bacterial community. Our second objective was to use RT-qPCR to systematically compare the *Wolbachia* dynamic titers in *C*Las uninfected and infected samples. The results of this study will provide a new version to understand the bacterial community between different color morphs of the ACP and how *C*Las influences this relationship.

## 2. Materials and methods

### 2.1. Insects

The *C*Las uninfected stock colony was originally collected from citrus groves in 2012 in Ganzhou, Jiangxi Province, China. The *C*Las infected ACP adults were obtained according to the previously described method, which was obtained through transferring the 7-day-old ACP adults on the HLB-infected plant *Citrus paradisi* Macf for 30 days (Ammar et al., 2011). The insects were kept at 26 ± 2°C with relative humidity (RH) of 40–50% and a photoperiod of 14:10 h (hr) (light: dark).

### 2.2. The sample and tissue collection

Adults were reared on *M. paniculata* seedlings in plastic cages (6 × 22 × 9 cm) under laboratory conditions of 26 ± 2°C, 40–50% relative humidity, and a 14:10 (light: dark) photoperiod until adulthood, when they were collected at 7-day-old as *C*Las uninfected samples. *C*Las uninfected 7-day-old ACP adults were kept and reared on HLB diseased plants for about 30 days before they were collected as *C*Las infected samples. We used the microscope (B301, OPTECN, Chongqing, China) to distinguish between blue-colored or gray-colored males and female adults. Each experimental group had four biological replicates, and every replicate had four ACP adults. The *C*Las uninfected and infected tissues were collected from previously described ACP adult treatments. The cuticle, gut, mycetome, and Malpighian tubule were dissected from the mixed-color morphs of ACP. The testis or ovary was collected from the mixed color morphs of the male or female ACP. All tissue samples were collected using the forceps (0203-4-PO, DUMONT, Jura, Switzerland) under a microscope (B301, OPTECN, Chongqing, China) on a glass slide with droplets of phosphate-buffered saline and washed three times with fresh phosphate-buffered saline. A total of four biological replicas comprised of pools of dissected tissues from over 200 ACP adults were collected.

We placed 7-day-old *C*Las uninfected ACP adults from *M. paniculata* to HLB-free hosts of *Citrus paradisi* Macf. for more than 30 days to investigate how the plants affect the ACP bacterial community, we chose *Wolbachia* as a representative bacteria to test how the plant species affect the ACP bacterial community. Each experimental group had six biological replicates, and every replicate had four mixed-gender ACP adults. All samples were stored in sterile tubes and preserved with 75% ethyl-alcohol at −80°C for downstream deoxyribonucleic acid extraction.

### 2.3. DNA extraction

DNA extraction from tissue samples was performed using the QIAGEN DNeasy Kit (QIAGEN, Hilden, Germany) according to the manufacturer’s specifications (Jiang et al., 2021). All of the DNA samples were quality-checked, and A260/A280 and A260/A280 values from 1.95 to 2.10 were used to qualify samples for further processing. DNA concentrations were quantified with a NanoDrop 2000 spectrophotometer (Thermo Fisher Scientific, Wilmington, DE, United States). All of the DNA extracts were stored at −20°C before polymerase chain reaction (PCR) was performed.

### 2.4. PCR amplification and sequencing of 16S rRNA amplicons

To assess the bacterial community composition of *C*Las-uninfected and *C*Las-infected samples from different color morphs and tissues of the ACP, 16S rRNA amplicon sequencing was conducted. Bacterial 16S rRNA gene fragments (V3-V4) were amplified from the extracted DNA samples using primers 338F (5’-ACTCCTACGGGAGGCAGCAG-3′) and 806R (5’-GGACTACHVGGGTWTCTAAT-3′) (Nan et al., 2016), and PCR was carried out in 20-μL reaction mixture containing 4 μl of 5 × FastPfu buffer, 2 μl of 2.5 mM dNTPs, 0.8 μl of each primer (5 μmol), 0.2 μl of Bovine albumin, 10 ng of template DNA. The PCR cycling parameters were 95°C for 3 min, 27 amplification cycles at 95°C for 30 s, 55°C for 30 s, and 72°C for 45 s, with a 10-min final extension at 72°C. Subsequently, PCR products were visualized on 2% agarose gel. High throughput sequencing was conducted on the Illumina Miseq platform and read lengths were determined using PE300 by Shanghai Majorbio Bio-pharm Technology Co. Ltd. (Shanghai, China).

### 2.5. Bioinformatics processing of amplicon datasets

Paired-end reads were merged using FLASH (v1.2.7) (Magoc and Salzberg, 2011), based on unique barcodes. Subsequently, reads were truncated by removing barcodes and adapter sequences and underwent quality-filtering using fastp (0.19.6) (Chen et al., 2018). Amplicon sequence variants (ASVs) were identified using the QIIME2 (version 2020.2) (Bolyen et al., 2019) pipeline. This process produced de-noised high-quality sequences generated by DADA2 with the single-nucleotide resolution based on sample error profiles (Callahan et al., 2016). Taxonomic assignments were made using the Naive Bayes consensus taxonomy classifier implemented in QIIME2 and the SILVA 16S rRNA database (v138). The alpha level diversities among host species were compared using the Shannon diversity index and Chao1index (Shannon, 1948). The processing steps for our 16S rRNA gene fragment library were performed on the free online platform Majorbio Cloud Platform.^1^

### 2.6. PCR amplified detection of *C*Las infections

To compare the difference of the acquired *C*Las ratios, the different color morph and gender adults were collected as the DNA samples. The same color and gender samples were as the one group. Each group had forty-four biological replicates, and each replicate had one adult sample. The extracted DNA samples using primers OI (5’-GCGCGTATCCAATACGAGCGGCA-3′) and OII (5’-GCCTCGCGACTTCGCAACCCAT-3′) (Jagoueix et al., 1996), and PCR was carried out in 20-μL reaction mixtures containing 10 μl of 2 × Taq buffer, 0.4 μl of each primer (5 μmol), 8.2 μl of sterile water, 1 μl of template DNA. The PCR cycling parameters were 94°C for 3 min, 34 amplification cycles at 94°C for 20 s, 58°C for 20 s, and 72°C for 80 s, with a 10-min final extension at 72°C. The sterile water was used as a negative control for all PCRs. Subsequently, PCR products were visualized on 2% agarose gel. If the sample had *C*Las-specific fragments detected as a band during gel electrophoresis, it was considered infected, whereas no detected band was considered an uninfected sample.

### 2.7. Construction of standards for qPCR

The *ftsZ* gene was the amplified sequence for building the *Wolbachia* quantitative real-time PCR standard (Supplementary Table S1). The obtained PCR products were purified and inserted into the pMDTM19-T Vector Cloning Kit (TaKaRa, Kusatsu, Japan) and used to transform Trans5α chemically competent cells, which were grown in a Luria-Bertani (L.B.) culture medium supplemented with 100 μg/ml ampicillin and 5-bromo-4-chloro-3-indolyl-β-D-galactopyranoside (X-GAL), following the manufacturer’s recommendations. Positive clones were isolated and cultivated in L.B. liquid medium supplemented with 100 μg/ mL ampicillin. The TaKaRa MiniBEST Plasmid Purification Kit Ver (TaKaRa, Kusatsu, Japan) was used to extract plasmids that would be subjected to PCR amplification using the specific primer sets developed for *Wolbachia* in a thermocycler set at 10 min at 95°C, followed by 20s at 95°C and 1 min at 60°C (35 cycles), followed by insert size verification on a 1% agarose gel under electrophoresis. Plasmids containing the correct insert size were used to produce a dilution standard curve. A series of ten dilutions containing 764 ng/μL-7.6 pg./μL of plasmid insert was amplified in triplicate using optimized cycling conditions for each primer set on a LightCycler® 96 PCR detection system (Roche, Basel, Switzerland) as follows: 95°C for 10 min, 40 cycles at 95°C for 10s, 60°C for the 20s, and 72°C for 20s, followed by a melting curve at 95°C for 10s, 65°C for 1 min, and 97°C for 1 s, cooling 37°C for 30s. The eluted DNA was quantified using a Thermo Scientific NanoDrop 2000c UV–Vis spectrophotometer (Thermo, Massachusetts, United States), and copy numbers were calculated by the following formula (Godornes et al., 2007):


$$\begin{array}{l} \mathrm{Copy number}/\mu l=\\ \frac{6.02\times 10^{23}\left(molecules/mole\right)\times DNA\;concentration\left(g/\mu l\right)\;}{Numberofbasepairs\times 660\;Daltons} \end{array}$$

where 6.022 × 10^23^ (molecules/mol) represented Avogadro’s constant, and 660 Da was the average weight of a single base pair (bp).

### 2.8. Statistical analyses

The significance of differences in alpha-level diversity was tested with an analysis of variance (ANOVA), followed by Tukey’s range test to assess the differences between the ACP samples, and beta-level diversity was assessed by the similarities test (ADONIS, replacement number 999 times for analysis). Both were integrated into the QIIME 2 pipeline version 2020.2 (Bolyen et al., 2019). The statistical significance of the *Wolbachia* dynamic titer in different color morphs and tissues of the *C*Las uninfected and infected ACP of qPCR data was analyzed by Student’s *t*-test. The *Wolbachia* dynamic titer change in *Citrus paradisi* Macf. of qPCR data was analyzed by ANOVA. The Shannon diversity and Chaol indices, symbiotic proportion were also compared with the non-parametric analysis (*, *p* < 0.05; **, *p* < 0.01; ***, *p* < 0.001; *p* > 0.05, N.S.: No Significance). Comparing the infected *C*Las rate used a *Z* test (*p* > 0.05, N.S.: No Significance). The data were analyzed using SPSS Version 21(IBM, Armonk, NY, United States). The Robust correlations with Spearman’s correlation coefficients >0.6 and false discovery rate corrected *p*-values <0.05 were used to construct networks. The visualizations were generated using the Gephi (Jacomy et al., 2014).

## 3. Results

### 3.1. 16S rRNA gene sequencing in different color morphs of *C*Las uninfected ACP adults

**Figure 1:**
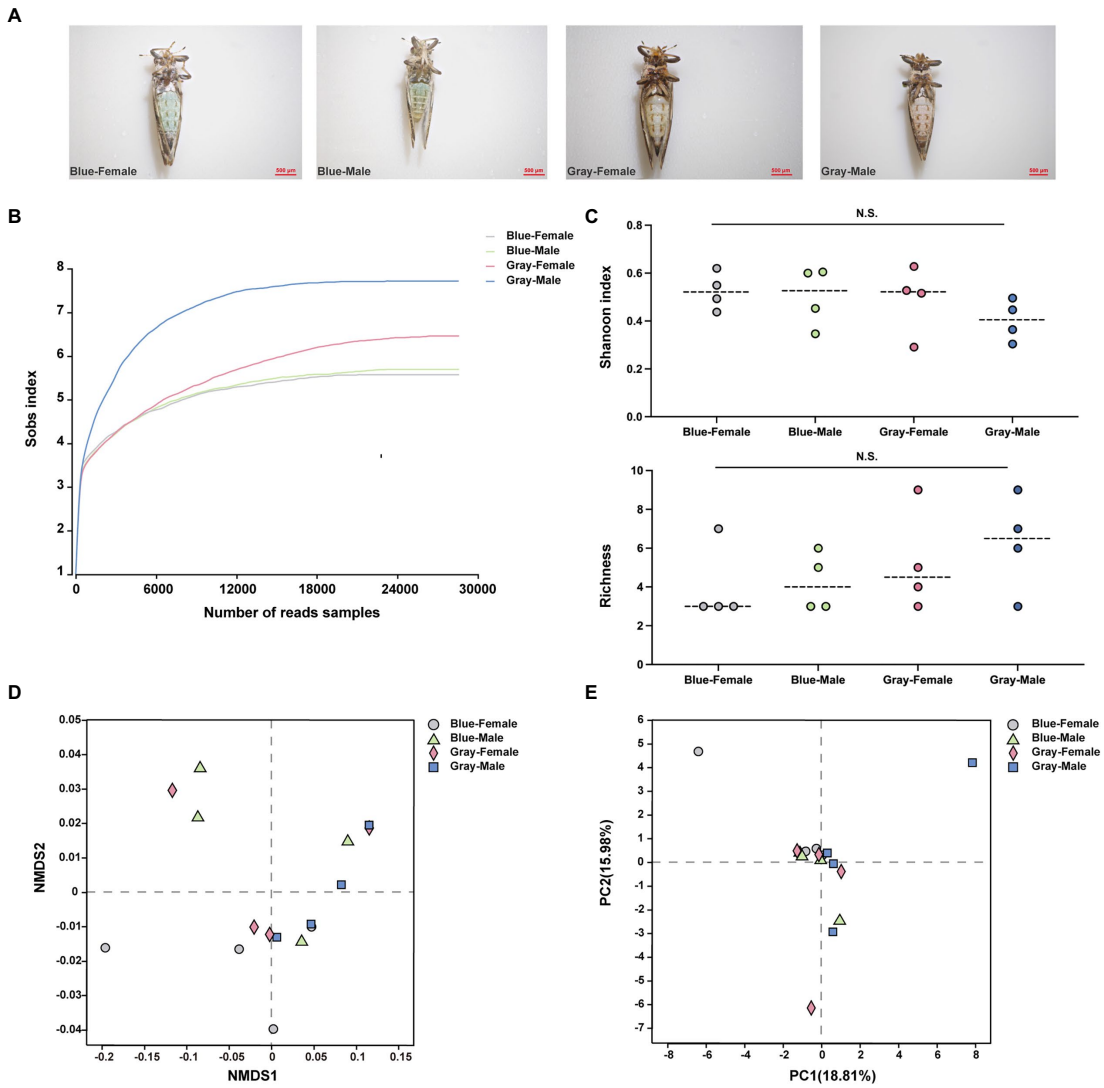
The alpha and beta analysis of different color morphs and genders of ACP adults. **(A)** Different color morphs and genders of ACP adults. **(B)** Rarefaction curves of read numbers in the different color morphs and genders of ACP. **(C)** Shannon index (up) and Chaol index (bottom) showed the bacterial community diversity and richness from different color morphs and genders of ACP adults. **(D)** Non-metric multidimensional scaling (NMDS) analysis. **(E)** Principal components analysis (PCA). The data sets were analyzed by the analysis of variance (ANOVA) followed by Tukey’s test, comparison of Shannon diversity and Chaol indices were by non-parametric analysis (*p* > 0.05 means no significant difference. N.S., no significance).

All sample data were obtained from blue and gray morphs of *C*Las uninfected ACP adults (Figure 1A), and each sample generated more than 28,000 reads (Figure 1B; Supplementary Table S2). Clustering analysis (97% similarity threshold) generated 37 ASVs, belonging to 20 families and five phyla. Overall, the phylum Proteobacteria was the most abundant in all samples (99%) (Supplementary Figure S1). The alpha level diversity indices were estimated to determine the bacterial community diversity (Shannon index) and richness (Chaol index) across the different color morphs and genders of ACP, and the results showed that there were no significant differences (*p* > 0.05; Figure 1C). At the beta-level analysis of diversity, Non-metric Multidimensional Scaling (NMDS) (*R* = −0.041, *p* = 0.596), Principal Component Analysis (PCA) (*R* = −0.059, *p* = 0.672), and Principal Co-ordinates Analysis (PCoA) (*R* = −0.041, *p* = 0.596) reflected the bacterial community of different color morphs and genders were not significantly different (Figures 1D,E; Supplementary Figure S2A). *Candidatus* Profftella and *Wolbachia* had a high proportion in different color morphs and genders of ACP samples (Supplementary Figure S2B,C). The four ASVs were shared among all groups (Supplementary Figure S2D). In the comparison of symbiotic relative proportions in different color morphs and genders, the results showed that the *Bosea* proportion between the gray-color female and male had a significant difference (*p* < 0.05), and other groups were not significantly different (Supplementary Figure S3).

### 3.2. The *Wolbachia* titer dynamic and the rate of the acquired *C*Las ratio in ACP adults

The *Wolbachia* titers in *C*Las uninfected ACP adults from *Murraya paniculata* to *Citrus paradisi* Macf. did not differ significantly after 30 days (Supplementary Figure 4A). Based on the PCR amplification and gel imaging analysis of the *C*Las uninfected ACP adult reared on the *C*Las infected plant for 30 days, the data showed that the different color morphs and genders of ACP acquired *C*Las at the same rate (Supplementary Figures S4B, S5).

### 3.3. 16S rRNA gene sequencing of *C*Las uninfected and infected ACP adults

**Figure 2:**
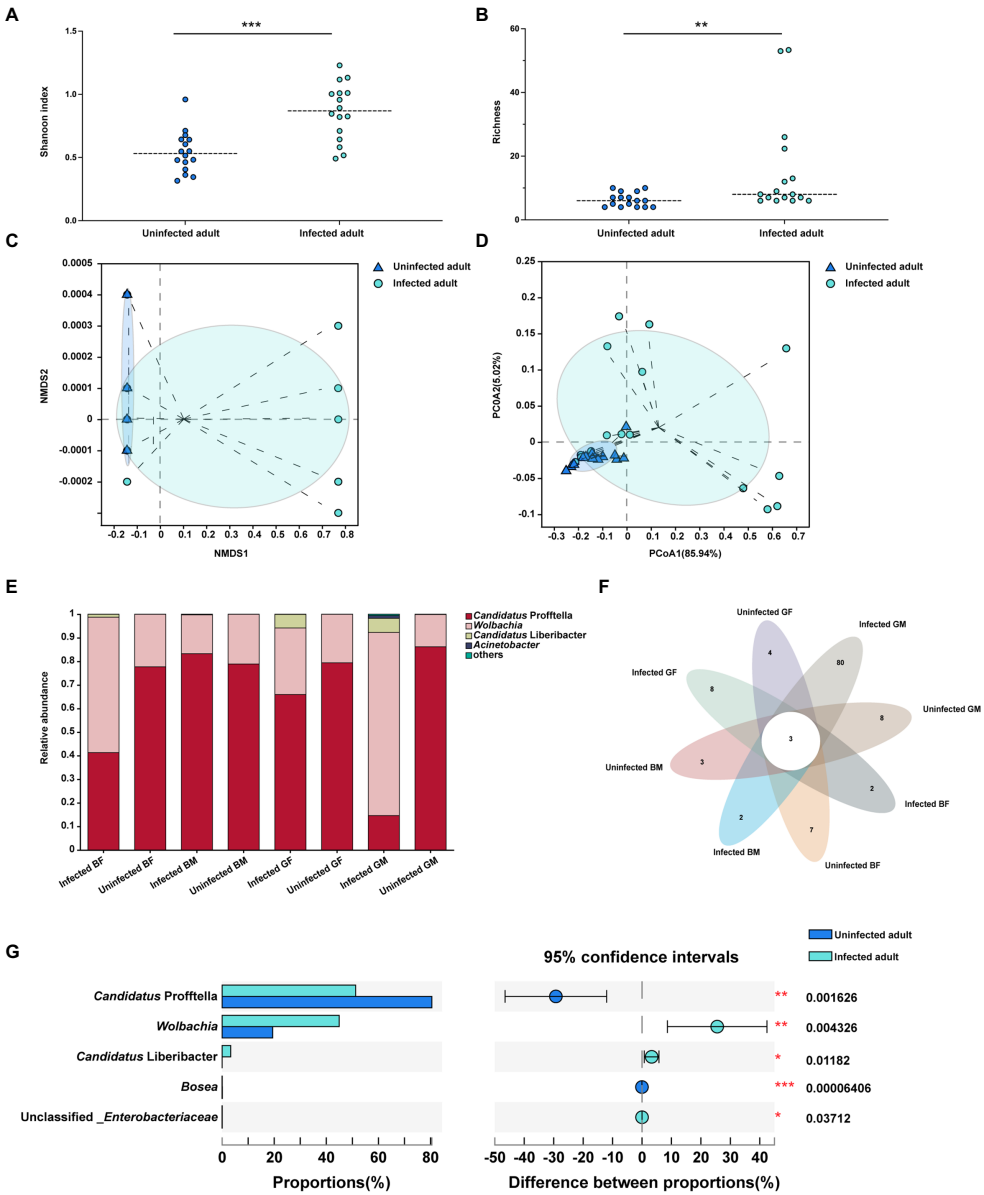
The alpha and beta level diversity analysis of different color morphs and genders of *C*Las uninfected and infected ACP adults. **(A)** Shannon diversity indices showed the bacterial community diversity of *C*Las uninfected and infected ACP adults. **(B)** Chaol indices showed the bacterial community richness of *C*Las uninfected and infected ACP adults. **(C)** Non-metric multidimensional scaling (NMDS) analysis. **(D)** Principal coordinates analysis (PCoA). **(E)** The bar plot of the bacterial proportion of *C*Las uninfected and infected ACP adults. **(F)** Core-Pan ASV presents the common and unique ASV of all samples in the petal diagram. **(G)** The bacterial proportion in *C*Las uninfected and infected ACP adults. The data were analyzed by the analysis of variance (ANOVA)-followed by Tukey’s test, comparison of Shannon diversity and Chaol indices and the difference of the bacterial proportions were by non-parametric analysis (*, *p* < 0.05; **, *p* < 0.01; ***, *p* < 0.001). BF, blue-female; BM, blue-male; GM, gray-male; GF, gray-female.

The data obtained from *C*Las infected ACP adults showed clean reads of 732,089 and a mean length of 404–425 bp for each sample. Each sample had more than 99% coverage of the bacterial taxa (Supplementary Table S3). According to the alpha-level diversity analysis, *C*Las infected ACP adult samples had significantly higher Shannon (*p* < 0.001) and Chaol (*p* < 0.01) indices than *C*Las uninfected adult samples (Figures 2A,B). Two clustering algorithms, including NMDS (stress <0.05, *p* = 0.004) and PCoA (*R*^2^ = 0.2491, *p* = 0.004) based on Bray-Curtis dissimilarities, were used to estimate the differences of the bacterial communities among *C*Las uninfected and infected adults at the ASV level. The results showed a divergence in the ASV-level clustering between *C*Las uninfected and infected adults (Figures 2C,D). The bar plot showed that *Candidatus* Profftella and *Wolbachia* were dominant symbionts in the *C*Las uninfected and infected conditions (Figure 2E). The petal diagram showed that common and unique ASVs were found in different color morphs and genders of the *C*Las uninfected and infected ACP adults, but the three ASVs were common in all samples (Figure 2F). Furthermore, the *C*Las uninfected adult samples had higher *Candidatus* Profftella (*p* < 0.01) and *Bosea* (*p* < 0.001) proportion than infected adult samples, but *Wolbachia* (*p* < 0.01), *Candidatus* Liberbacter (*p* < 0.05) and unclassified_*Enterobacteriaceae* (*p* < 0.05) had higher proportions than *C*Las uninfected adult samples (Figure 2G).

### 3.4. 16S rRNA gene sequencing in different tissues of *C*Las uninfected and infected of ACP adults

**Figure 3:**
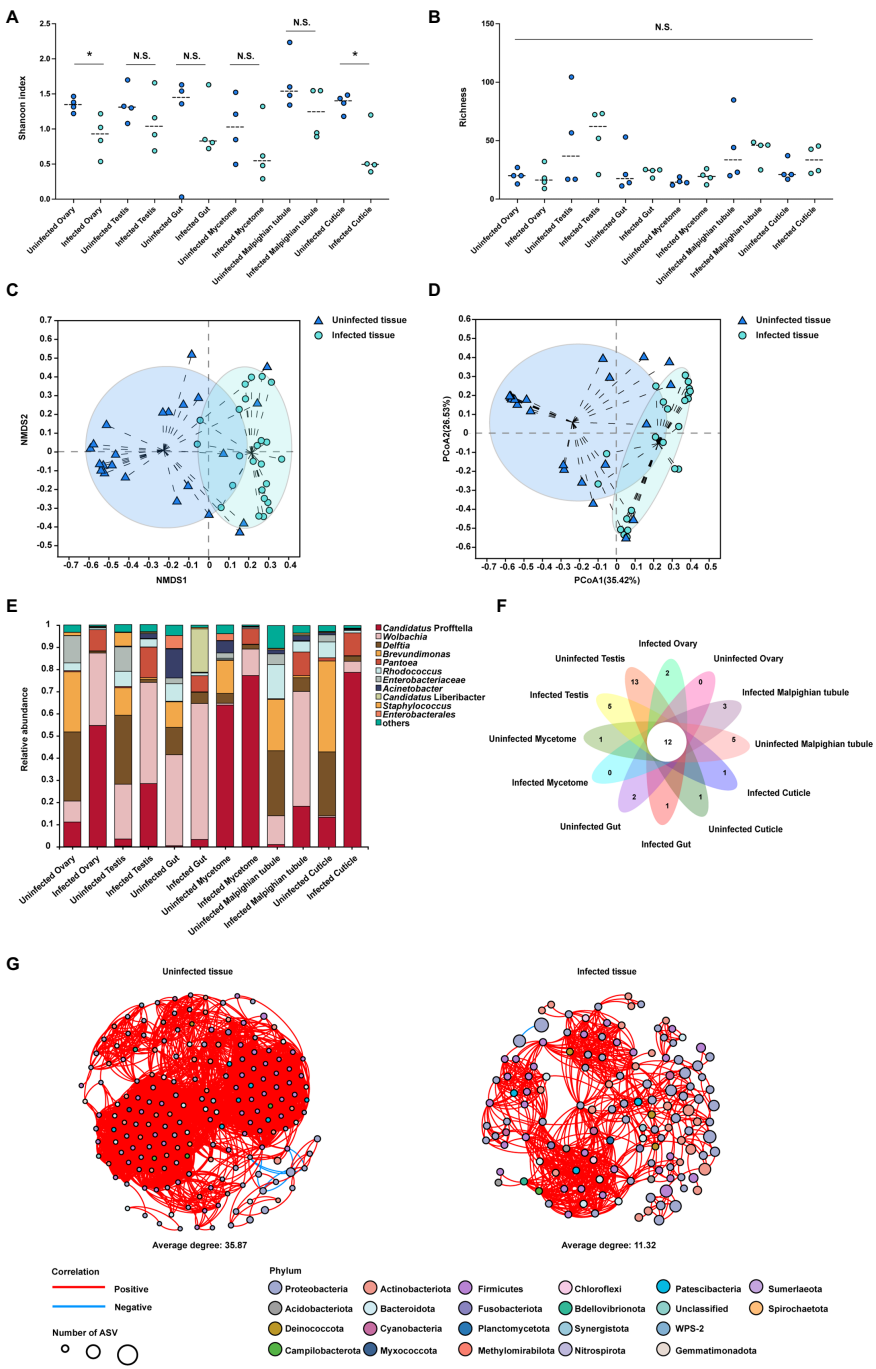
The alpha and beta diversity analysis in the tissues of *C*Las uninfected and infected ACP. **(A)** Shannon’s diversity index showed bacterial community diversity in different tissues of *C*Las uninfected and infected ACP. **(B)** The Chaol index showed bacterial community richness in different tissues of *C*Las infected ACP. **(C)** Non-metric multidimensional scaling (NMDS) analysis. **(D)** Principal components analysis (PCA). **(E)** The bar plot of the bacterial proportion in the tissues of *C*Las uninfected and infected ACP. **(F)** Core-Pan ASV presented the common and unique ASV of all samples in the petal diagram. **(G)** The bacterial network between different tissues of *C*Las uninfected and infected ACP. Edges represent significant Spearman correlations (*ρ* > |0.6|, *p* < 0.05). Light blue and red lines represent significant negative and positive correlations, respectively. The size of the points indicates the number of ASVs in each bacterial community. The data were analyzed by the analysis of variance (ANOVA) followed by Tukey’s test, comparisons of Shannon diversity and Chaol index were by non-parametric analysis (*, *p* < 0.05; *p* > 0.05 means no significant difference. N.S., no significance).

The data obtained from different tissues is presented in (Supplementary Figure S6A–F), and the total number of clean reads was 3,039,337, with a mean length of 410–428 bp for each sample. Each sample had more than 99% coverage of the bacterial taxa (Supplementary Table S4). Summary analysis from the QIIME2 pipeline revealed 625 ASVs that belonged to 138 families and 22 phyla. Tissues including cuticle, ovary and testis from the *C*Las infected samples had higher *Candidatus* Profftella proportion than uninfected samples. Malpighian tubule, mycetome and gut from the *C*Las infected samples had higher *Wolbachia* proportion than uninfected samples. The alpha level diversity analysis using Shannon diversity indices revealed that the ovary and cuticle were decreased in the *C*Las infected condition compared to the *C*Las uninfected condition (*p* < 0.01), other tissues showed no significant difference (*p* > 0.05; Figure 3A), and the Chaol index showed no significant difference (*p* > 0.05; Figure 3B). Two clustering algorithms, including NMDS (stress <0.2, *p* = 0.001) and PCoA (*R*^2^ = 0.2226, *p* = 0.001) based on Bray-Curtis dissimilarities, were used to estimate the differences of the bacterial communities among different tissues of *C*Las uninfected and infected ACP at the ASV level. The results showed a divergence in the ASV level clustering among different tissues (Figures 3C,D). The bar plot showed that the bacterial community proportion had changed between the tissues of *C*Las uninfected and infected ACP (Figure 3E). For example, in the Malpighian tubule and mycetome, the *Wolbachia* proportion increased compared to the uninfected condition. Compared to the uninfected condition, the *Candidatus* Profftella proportion had increased in the testis, gut, ovary, and cuticle. However, some bacterial proportions were lower when compared to uninfected conditions. For example, the *Delftia* proportion had decreased in the Malpighian tubule, testis, ovary, and cuticle (Supplementary Figure S7). The petal diagram showed the common and unique ASVs numbers in the tissues of *C*Las uninfected and infected ACP and the 12 ASVs in common within all samples (Figure 3F). The bacterial network in tissues of *C*Las uninfected ACP (average degree: 35.87) was more complicated than in infected condition (average degree: 11.32) (Figure 3G). The number of edges in tissues of *C*Las uninfected ACP (3,327 positives and nine negatives) was higher than that in tissues of *C*Las infected ACP (769 positives and one negative).

### 3.5. The *C*Las altered the *Wolbachia* dynamic in different color morphs and tissues of ACP adults

**Figure 4:**
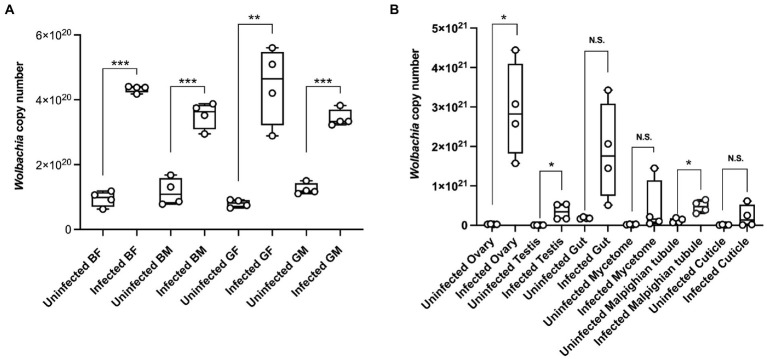
The *Wolbachia* dynamic titer in different color morphs and tissues of the *C*Las uninfected and infected ACP. **(A)** Different color morphs and genders of ACP adults. **(B)** Different tissues of ACP. Comparison of the difference of qPCR data was analyzed by Student’s *t*-test (*, *p* < 0.05; **, *p* < 0.01; ***, *p* < 0.001, *p* > 0.05 means no significant difference. N.S., no significance). BF, blue-female; BM, blue-male; GM, gray-male; GF, gray-female.

We used quantification to determine *Wolbachia* titer dynamics in different color morphs and tissues of *C*Las uninfected and infected ACP adults. The results showed that the *Wolbachia* titers were significantly higher in *C*Las infected adults than in uninfected adults (Figure 4A). At the same time, the *Wolbachia* titers in the testis, ovary, and Malpighian tubules of *C*Las infected adults were significantly higher than in the uninfected condition (Figure 4B).

## 4. Discussion

### 4.1. The bacterial community in different color morphs of ACP adults

Previous studies showed that many factors could impact the bacterial diversity of arthropods, including insect genders, developmental stages, host plants, and geographical locations (Gusmão et al., 2007; Koch et al., 2013; McFrederick et al., 2014; Martinson et al., 2017; Adair et al., 2018; Su et al., 2021). In this study, the samples were selected at the same developmental stage and reared on the same plant host species under laboratory conditions, which can exclude variable factors (development stages, host species, and environmental factor differences) from affecting the results. The data demonstrated that the bacterial alpha-level diversity between the different color morphs and genders of ACP adults showed no significant difference. The beta-level showed the bacterial community composition of the different color morphs and genders of ACP were not significantly different. *Candidatus* Profftella and *Wolbachia* had higher proportions in different color morphs and genders of the ACP. Congruently, the different color morphs of ACP acquiring *C*Las at the same rate had no significant differences. Therefore, based on our findings, we propose that the different color morphs of ACP displayed distinct characteristics that had little connect with the bacterial community.

### 4.2. *C*Las affecting the bacterial community between different color morphs and tissues of the ACP adults

Arthropods, or in our case, ACP, have different morphs and tissues which harbor diverse bacterial communities. But, we still have little understanding of how *C*Las infections affect the bacterial community between different morphs and tissues of ACP. This study investigated the bacterial communities in different morphs and tissues of *C*Las uninfected and infected ACP adult, and the results showed that *C*Las could increase the entire bacterial community’s diversity and richness within the whole body of ACP. However, the proportion of different bacterial genera had changed. The proportion of *Candidatus* Profftella and *Bosea* was higher in the uninfected condition than in the infected condition. Meanwhile, the *Wolbachia* and *Enterobacteriaceae* proportions in the uninfected condition were lower than in the infected condition. Additionally, some symbionts were only found in certain ACP tissues. For example, *Leptotrichia* and *Veillonella* were detected in the testis, ovary, and Malpighian tubule. The *Exiguobacterium* was present in the ovary, mycetome, and Malpighian tubule. *Pseudomonas* was found in the gut, mycetome, testis, and Malpighian tubule (Ateyyat et al., 2010; Chavshin et al., 2012, 2014). However, some symbionts were detected in all ACP tissue samples, such as *Candidatus* Profftella, *Wolbachia*, and *Acinetobacter*, which were also present in other insects (Snyman et al., 2016; Chung et al., 2017; Hussain et al., 2017). *C*Las could reduce the diversity of the ovary and cuticle bacterial communities compared to the uninfected condition. At the same time, *C*Las could make the bacterial correlation network between the different tissues simpler than it is in an uninfected condition.

### 4.3. The *C*Las affecting the *Wolbachia* titer in different color morphs and tissues of ACP adults

Previous studies showed that *Wolbachia,* as a facultative endosymbiont, can affect host reproduction (Engelstädter and Hurst, 2009; Berasategui et al., 2017; Pan et al., 2017; Ju et al., 2020). However, how *Wolbachia* participates in the ACP study is still in its early stages. In our study, the *Wolbachia* titers showed a significant increase in infected adult samples compared to uninfected adult samples. The gut was considered an important tissue for *C*Las to invade the ACP host. Previous research found that the *Wolbachia* titer increased when the ACP was exposed to extreme high-temperature conditions (Hussain et al., 2017; Jiang et al., 2021). This phenomenon implies that *Wolbachia* could play an important role within the host ACP. Our experimental results provide novel information within our field of study when examining *Wolbachia* in ACP.

## 5. Conclusion

This study partially fills the gap in the ACP bacterial community characteristics of *C*Las uninfected and infected individuals with different color morphs and tissues. The study combines previous studies to form a hypothesis that the different color morphs of ACP showed different characteristics that could be derived from the bacterial community diversity. Our results indicated that these characteristics did not relate to the bacterial community. On the other hand, we discovered that *C*Las was a significant external factor that could influence the ACP bacterial community. In summary, this study offers a systematic survey of the bacterial communities in different colors morphs and tissues of ACP in a standardized manner.

## Data availability statement

The datasets presented in this study can be found in online repositories. The names of the repository/repositories and accession number(s) can be found in the article/Supplementary material.

## Author contributions

R-XJ and TC: writing the original draft. R-XJ, FS, WD, and J-JW: methodology, software, data curation, and validation. R-XJ: psyllid sampling and experiment. FS, WD, H-BJ, TC, and J-JW: conceptualization, supervision, funding acquisition, and review and editing. All authors contributed to the article and approved the submitted version.

## Funding

This research was supported by the National Key R&D Program (2021YFD1400802), China Agriculture Research System (CARS-26) and the 111 Project (B18044).

## Conflict of interest

The authors declare that the research was conducted in the absence of any commercial or financial relationships that could be construed as a potential conflict of interest.

## Publisher’s note

All claims expressed in this article are solely those of the authors and do not necessarily represent those of their affiliated organizations, or those of the publisher, the editors and the reviewers. Any product that may be evaluated in this article, or claim that may be made by its manufacturer, is not guaranteed or endorsed by the publisher.

## Footnotes

^1^ cloud.majorbio.com
