# Supplementary material for: Candidatus Liberibacter asiaticus: An important factor affecting bacterial community composition and Wolbachia titers in Asian citrus psyllid
Source: Front Microbiol. 2023 Feb 7;14:1109803. doi: 10.3389/fmicb.2023.1109803 (PMC9941154; doi:10.3389/fmicb.2023.1109803)
Supplement: Supplementary file 1 [file Data_Sheet_1.docx]

Supplementary Material


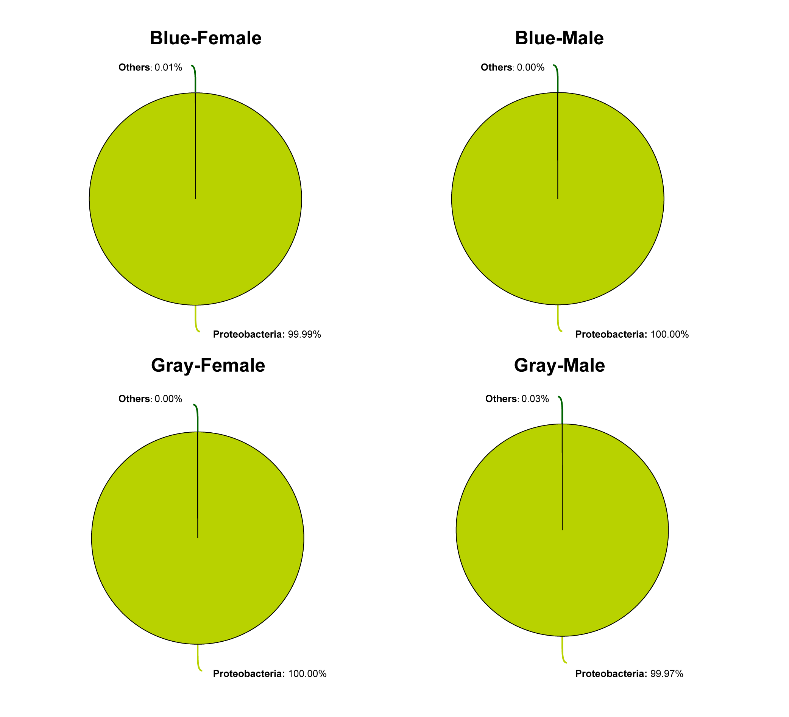


Supplementary Figure 1. Pie chart depicting the relative abundance of bacteria of CLas uninfected ACP adult samples.


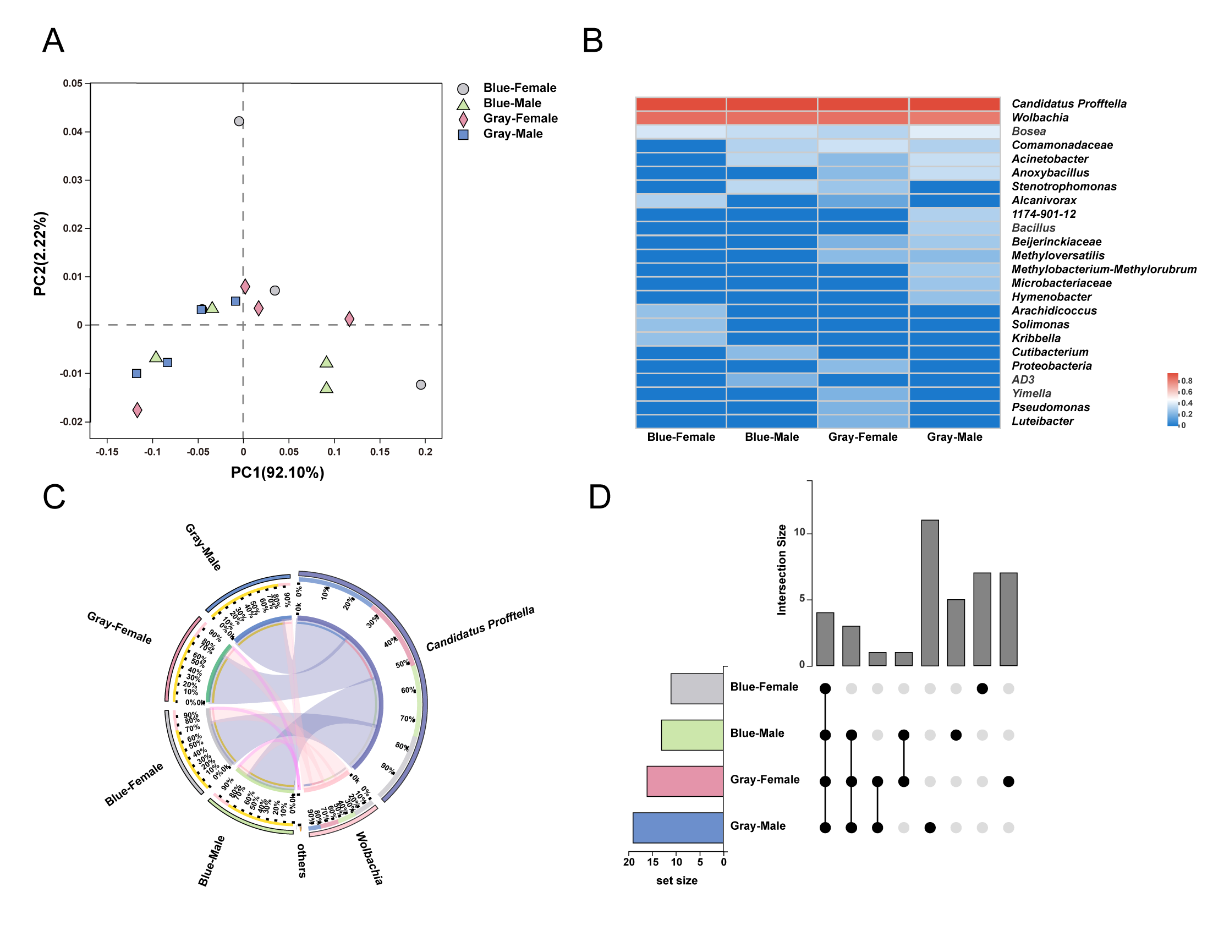


**Supplementary Figure 2.** Rarefaction curves of beta diversity indexes of ACP adults. **(A)** Principal coordinates analysis (PCoA). **(B-C)** The bacterial community composition of the different color morphs and genders of ACP. **(D)** Setup diagram of shared and unique ASVs numbers observed in different abdominal colors and genders of ACP. The data were analyzed by the analysis of variance (ANOVA) followed by Tukey’s test.


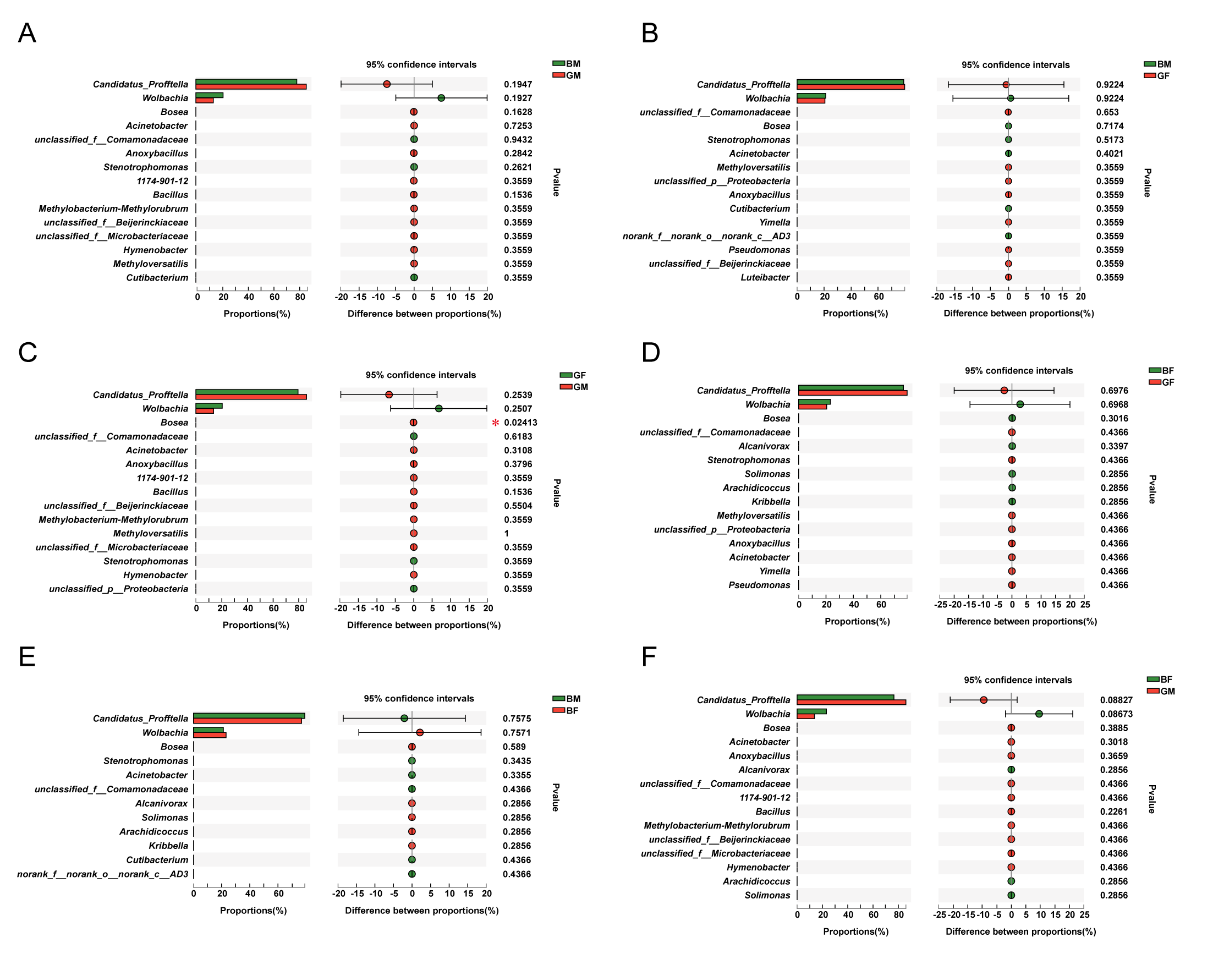


**Supplementary Figure 3.** Comparison of the bacterial community with different color morphs and genders of ACP. BF: Blue-Female, BM: Blue-Male, GM: Gray-Male, GF: Gray-Female. The data was analyzed by non-parametric analysis (*, *p* < 0.05).


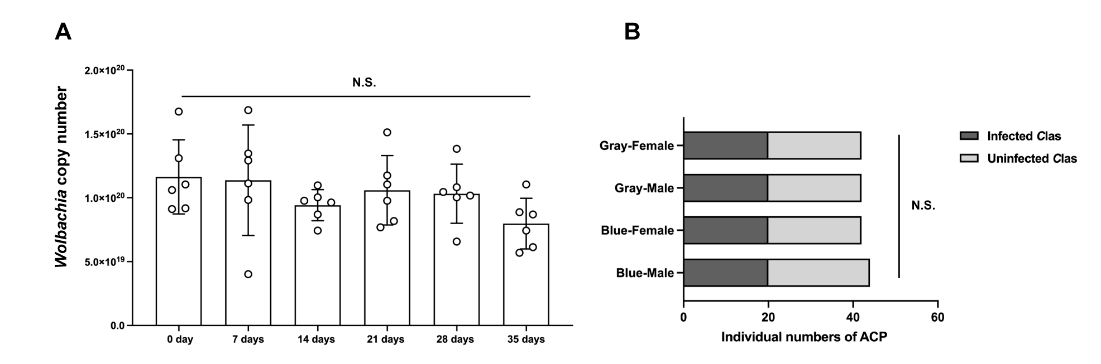


**Supplementary Figure 4.** Comparing the different color morphs and genders of ACP acquired the *C*Las ratio. **(A)** The *Wolbachia* titer dynamic change in *Citrus paradisi Macf*. **(B)** The different color morphs and genders of ACP acquiring the rate of *C*Las. The comparison of the *Wolbachia* titer was analyzed by ANOVA (*p* > 0.05 means no significant difference. N.S.: No Significance) and comparing the difference of ACP acquiring the rate of *C*Las by *Z* test (*p* > 0.05 means no significant difference. N.S.: No Significance).


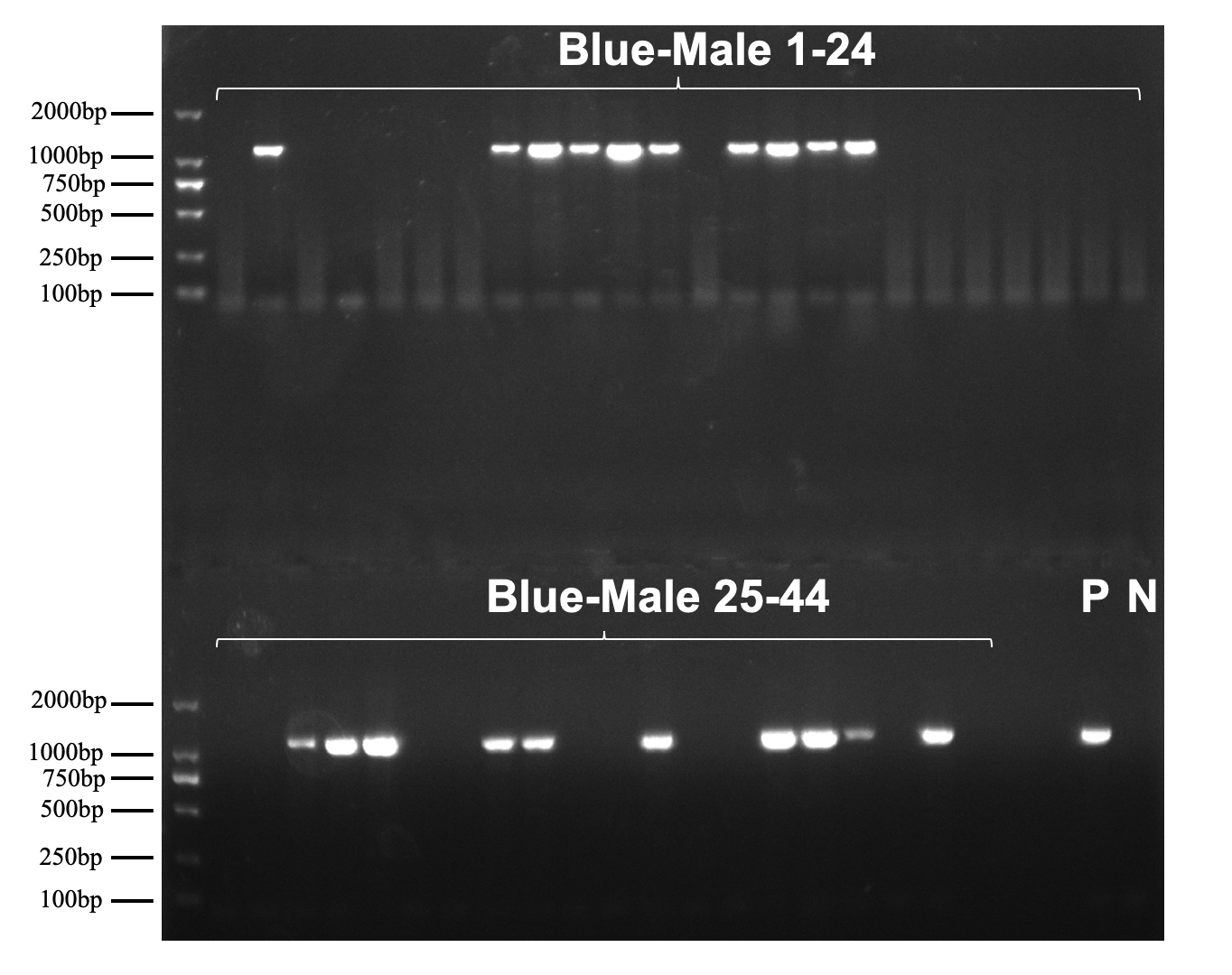

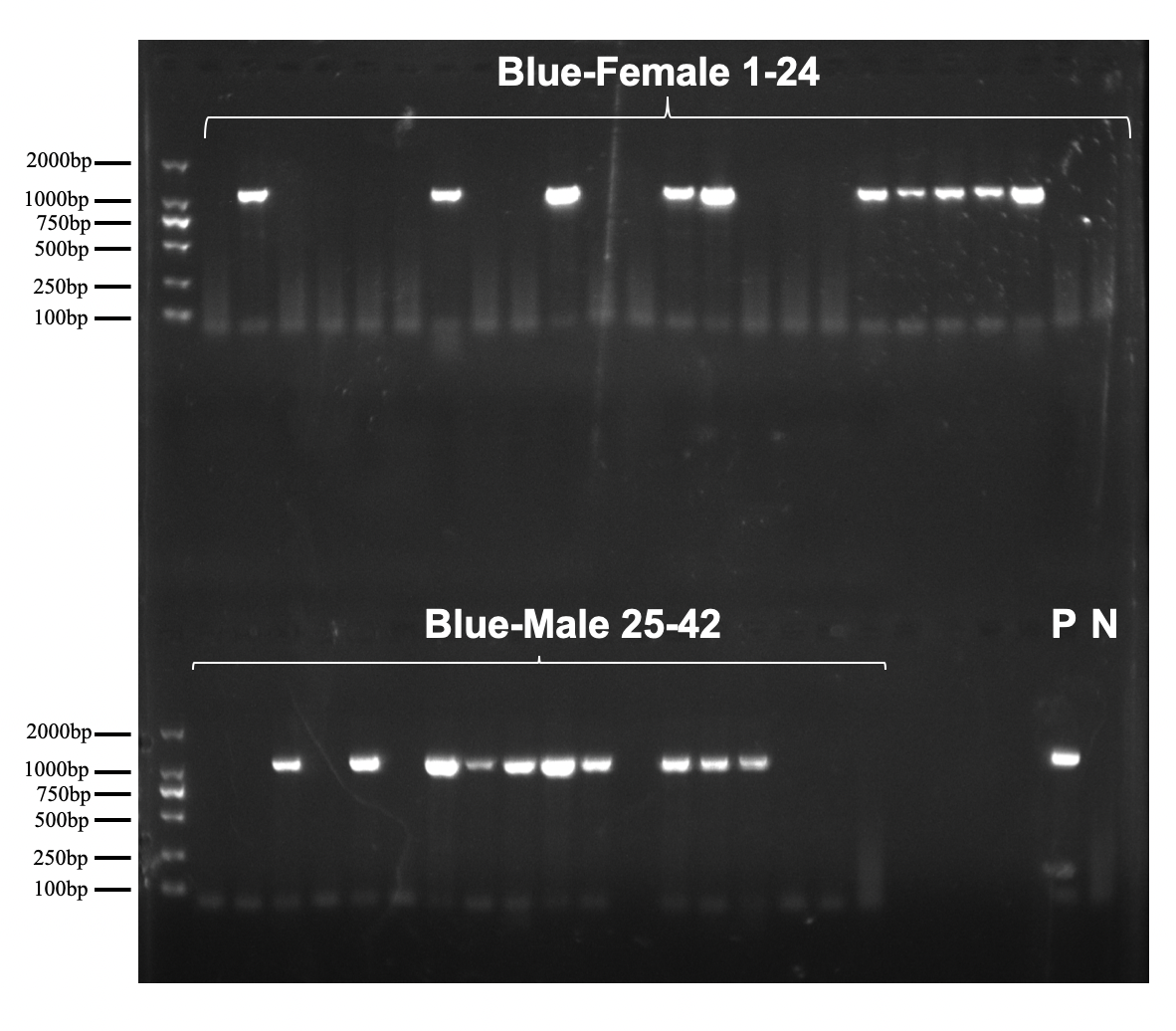

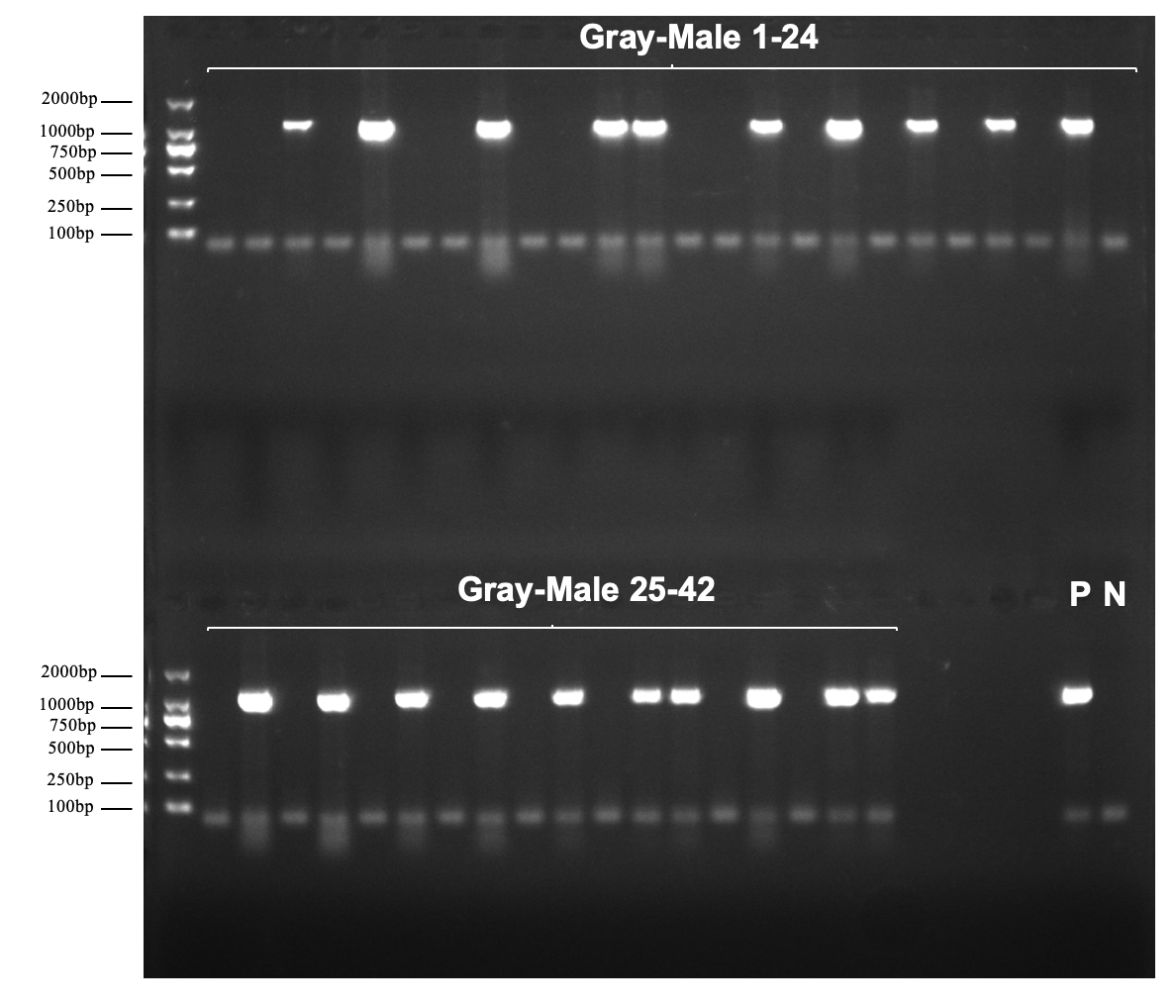

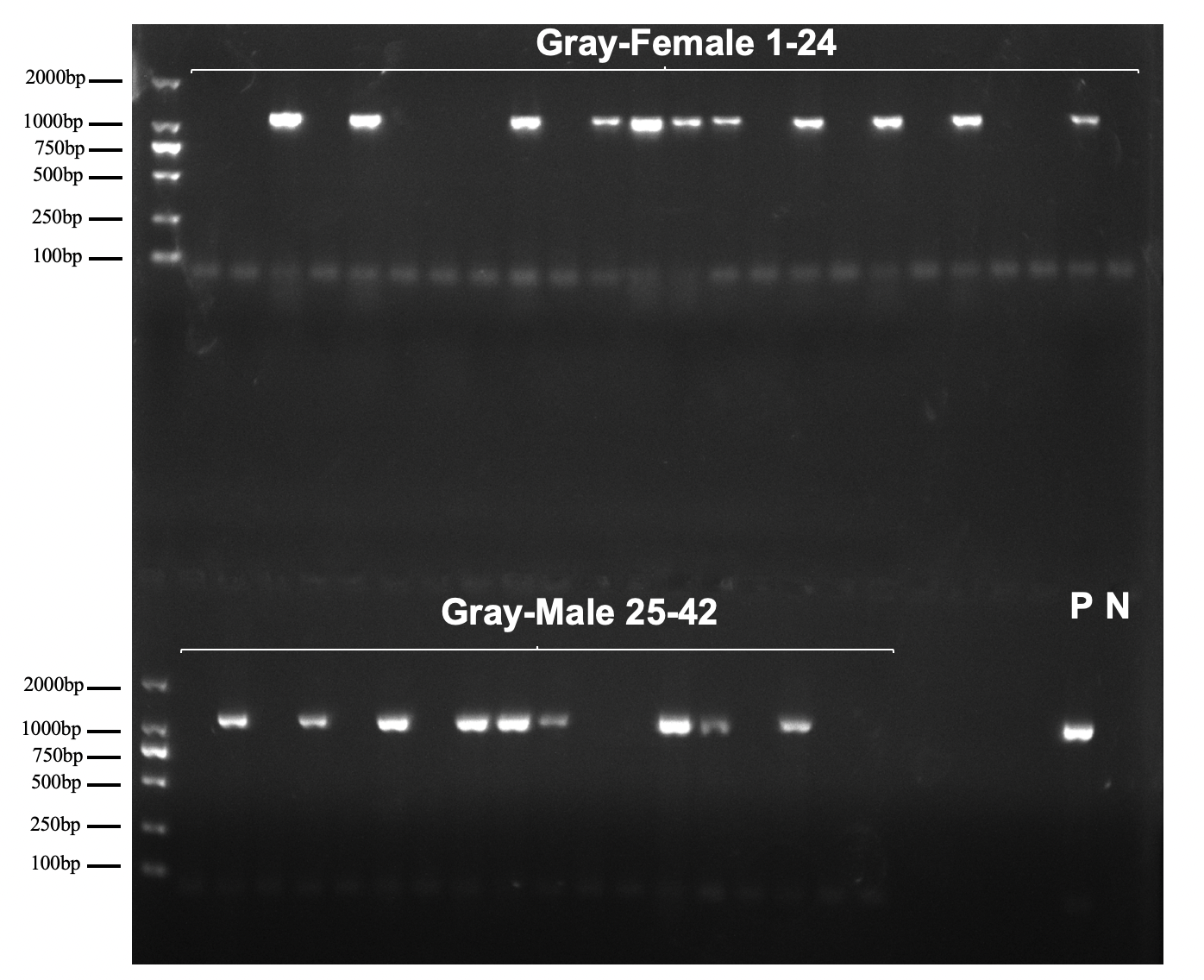


Supplementary Figure 5. PCR detection of *C*Las-specific sequence in different color morphs and genders of ACP. P: Positive control. N: Negative control.


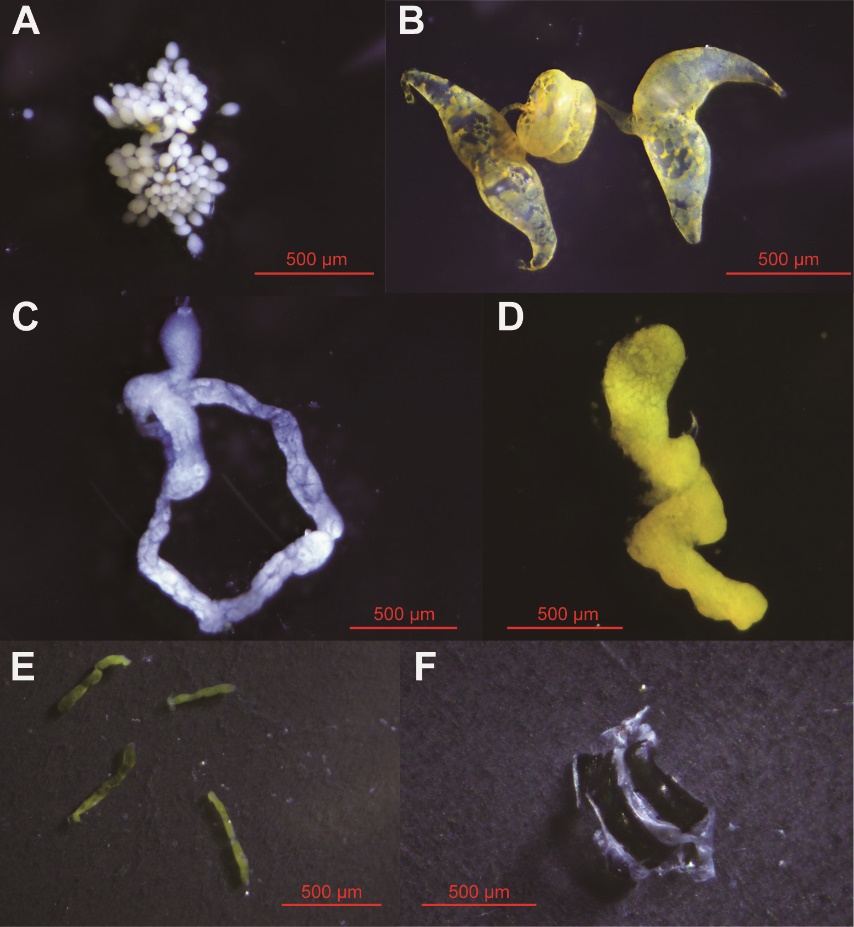


**Supplementary Figure 6.** The different tissues of ACP. **(A)** Ovary. **(B)** Testis. **(C)** Gut. **(D)** Mycetome. **(E)** Malpighian tubule. **(F)** Cuticle.


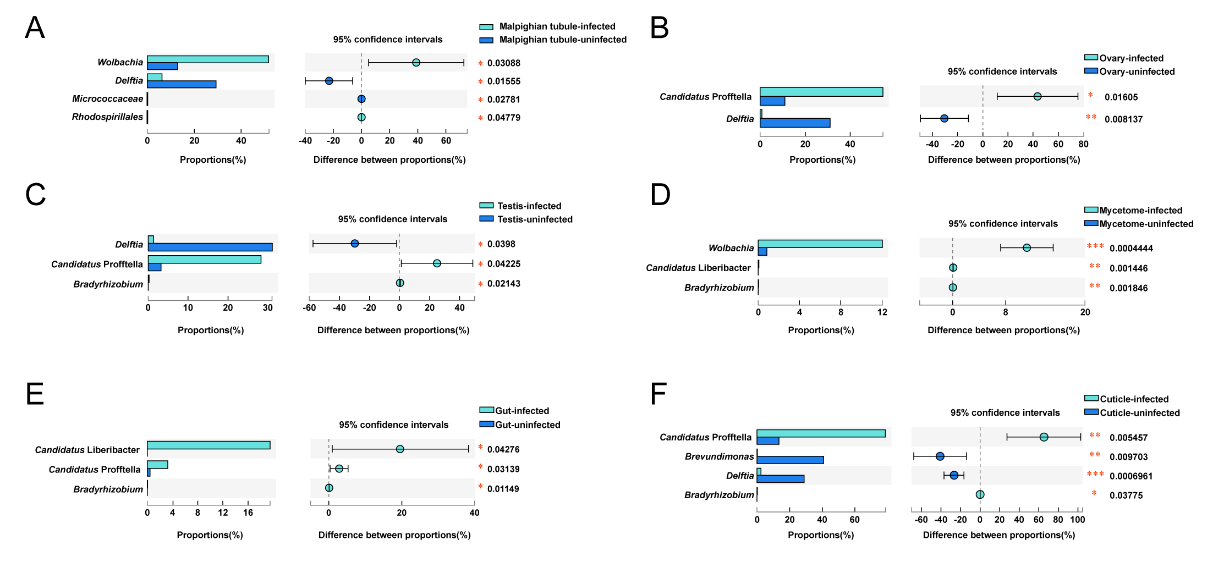


**Supplementary Figure 7.** Comparison of the bacterial community indifferent tissues of *C*Las uninfected and infected of ACP. Comparison of the different bacterial proportion was analyzed by non-parametric analysis. (*, *p* < 0.05; **, *p* < 0.01; ***, *p* < 0.001).

**Supplementary Table 1.** Primer sequences are used for quantitative real-time PCR (RT-qPCR).

| Target species | Target genes | Assay tape | Amplicon size (bp) | Primer sequence |
| --- | --- | --- | --- | --- |
| *Wolbachia* | *ftsZ*:KY658590.1 | SYBR | 110 | AGCAGCCAGAGAAGCAAGAG |
|  |  |  |  | TACGTCGCACACCTTCAAAA |

**Supplementary Table 2.** The 16S rRNA gene sequencing data from different color morphs and genders of *C*Las uninfected ACP.

| Sample | Sobs | ACE | Chaol | Shannon | Simpson | Coverage |
| --- | --- | --- | --- | --- | --- | --- |
| Blue-Female | 4.0000 | 3.2500 | 4.0000 | 0.4849 | 0.6910 | 0.9999 |
| Blue-male | 6.0000 | 6.0000 | 6.0000 | 0.4674 | 0.7091 | 0.9999 |
| Gray-Female | 5.2500 | 6.5861 | 5.0000 | 0.5275 | 0.6589 | 0.9999 |
| Gray-Male | 6.2500 | 6.2500 | 6.2500 | 0.4029 | 0.7644 | 0.9999 |

Data were shown as the mean data with four replicates, indicating ACP adult of blue or gray, female or male, respectively.

**Supplementary Table 3.** The 16S rRNA gene sequencing data from different color morphs and genders of *C*Las infected ACP.

| Sample | Sobs | ACE | Chaol | Shannon | Simpson | Coverage |
| --- | --- | --- | --- | --- | --- | --- |
| Blue-Female | 7.0000 | 7.0000 | 7.0000 | 0.9047 | 0.5096 | 1.0000 |
| Blue-male | 8.2500 | 8.3381 | 8.2500 | 0.6036 | 0.6944 | 1.0000 |
| Gray-Female | 8.0000 | 5.0250 | 8.5000 | 1.0057 | 0.4736 | 1.0000 |
| Gray-Male | 31.3333 | 36.6264 | 33.8889 | 0.2921 | 0.0877 | 0.9998 |

Data were shown as the mean data with four replicates, indicating ACP adult of blue or gray, female or male, respectively.

**Supplementary Table 4.** The 16S rRNA gene sequencing data in different tissues of *C*Las uninfected and infected ACP.

| Sample | Sobs | ACE | Chaol | Shannon | Simpson | Coverage |
| --- | --- | --- | --- | --- | --- | --- |
| Cuticle-uninfected | 29.6364 | 31.0970 | 30.3700 | 1.36500 | 0.35305 | 0.9999 |
| Cuticle-infected | 32.7500 | 34.8778 | 33.5910 | 0.64556 | 0,70677 | 0.9999 |
| Gut-uninfected | 26.9333 | 27.8934 | 27.3824 | 1.31969 | 0.39630 | 0.9999 |
| Gut-infected | 22.7500 | 24.9466 | 23.1696 | 1.00190 | 0.49388 | 0.9999 |
| Mycetome-uninfected | 27.9412 | 29.3839 | 28.7204 | 1.32608 | 0.37675 | 0.9999 |
| Mycetome-infected | 18.7500 | 20.6086 | 19.25 | 0.67641 | 0.65883 | 0.9999 |
| Testis-uninfected | 31.3333 | 32.3639 | 32.1139 | 1.36749 | 0.35340 | 0.9999 |
| Testis-infected | 48.5000 | 54.5422 | 54.5889 | 1.10599 | 0.47147 | 0.9999 |
| Malpighian tubule-uninfected | 25.9412 | 26.6947 | 26.3227 | 1.39127 | 0.35153 | 0.9999 |
| Malpighian tubule-infected | 40.0000 | 42.5383 | 41.4688 | 1.23169 | 0.42590 | 0.9999 |
| Ovary-uninfected | 20.0000 | 20.2861 | 20.0000 | 1.34524 | 0.34759 | 0.9999 |
| Ovary-infected | 18.0000 | 19.3851 | 18.4250 | 0.90300 | 0.47775 | 0.9999 |

Data were shown as the mean data with four replicates, indicating psyllid adult different tissues, respectively.
